# Multi-Staged Regulation of Lipid Signaling Mediators during Myogenesis by COX-1/2 Pathways

**DOI:** 10.3390/ijms20184326

**Published:** 2019-09-04

**Authors:** Chenglin Mo, Zhiying Wang, Lynda Bonewald, Marco Brotto

**Affiliations:** 1 Bone-Muscle Research Center, College of Nursing and Health Innovation, The University of Texas-Arlington, Arlington, TX 76019, USA; 2 Indiana Center for Musculoskeletal Health, School of Medicine, Indiana University, Indianapolis, IN 46202, USA

**Keywords:** Cyclooxygenase, skeletal muscle, myogenic differentiation, lipidomics

## Abstract

Cyclooxygenases (COXs), including COX-1 and -2, are enzymes essential for lipid mediator (LMs) syntheses from arachidonic acid (AA), such as prostaglandins (PGs). Furthermore, COXs could interplay with other enzymes such as lipoxygenases (LOXs) and cytochrome P450s (CYPs) to regulate the signaling of LMs. In this study, to comprehensively analyze the function of COX-1 and -2 in regulating the signaling of bioactive LMs in skeletal muscle, mouse primary myoblasts and C2C12 cells were transfected with specific COX-1 and -2 siRNAs, followed by targeted lipidomic analysis and customized quantitative PCR gene array analysis. Knocking down COXs, particularly COX-1, significantly reduced the release of PGs from muscle cells, especially PGE_2_ and PGF_2α_, as well as oleoylethanolamide (OEA) and arachidonoylethanolamine (AEA). Moreover, COXs could interplay with LOXs to regulate the signaling of hydroxyeicosatetraenoic acids (HETEs). The changes in LMs are associated with the expression of genes, such as *Itrp1* (calcium signaling) and *Myh7* (myogenic differentiation), in skeletal muscle. In conclusion, both COX-1 and -2 contribute to LMs production during myogenesis in vitro, and COXs could interact with LOXs during this process. These interactions and the fine-tuning of the levels of these LMs are most likely important for skeletal muscle myogenesis, and potentially, muscle repair and regeneration.

## 1. Introduction

Skeletal muscle myogenesis, such as muscle regeneration after injury, is a biological process critical for maintaining a functional musculoskeletal system. Myogenesis generally consists of several consecutive stages, including activation of satellite cells, proliferation of myoblasts, myogenic differentiation, and fusion into multinucleated myocytes that can later become fully mature and long, differentiated muscle cells, sometimes referred to as muscle fibers [1]. This process is highly coordinated, and many factors have been shown to be involved in the regulation of myogenesis [2].

Prostaglandins (PGs) are a group of lipid mediators (LMs) playing important roles in various physiological and pharmacological processes, such as fever, inflammation, reproductive function, tissue regeneration, and myogenesis [3,4,5,6]. In skeletal muscle, PGE_2_ and PGF_2α_, are the two most important PGs. PGE_2_ has been shown to enhance myoblast proliferation and differentiation [4,7], and PGF_2α_ is able to promote muscle cell survival and fusion [8,9].

PGs are derived from arachidonic acid (AA) through the activities of a series of specific enzymes. Cyclooxygenases (COXs), including COX-1 and -2, are the rate-limiting enzymes during this process. Generally, COX-1 is constitutively expressed in most cells, while COX-2 is inducible in a variety of pathological situations, such as inflammation and cancer development [10,11]. In skeletal muscle, the knowledge of COXs derives mostly from the studies of COX-2. In muscle repair or regeneration models, COX-2 knockout mice had delayed recovery from muscle injury, suggesting that COX-2 and the downstream PGs from this pathway could be important in regenerative myogenesis, especially in the early inflammatory phase of muscle regeneration for activation of neutrophils, macrophages, and satellite cells [12,13]. However, in this model, the roles of COX-1 and -2 in myogenic processes after the inflammation phase have not been defined. Moreover, in the hind limb suspension mouse model, the induction of COX-2 is essential for muscle recovery from the atrophy caused by unloading [14].

In addition to COXs, AA is also the substrate for lipoxygenases (LOXs) [15] and cytochromes P450 (CYPs) [16]. The metabolites via these two pathways include leukotrienes and hydroxy eicosatetraenoic acids, which are biological activators of intracellular signaling [17,18]. To our knowledge, the interactions between COXs, LOXs, and CYPs have not been studied in skeletal muscle. The changes in the functionalities of COXs would cause indirect effects resulting in modified activities of LOX and/or CYPs.

In this study, we investigated the functional relevance of COX-1 and -2 in myogenesis from myoblasts to the development of multi-nucleated myotubes in both C2C12 cells and mouse primary myoblasts. Since selective inhibitors of COX could reduce the production of PG through COX-independent pathways and could be unselective under certain conditions [19], in the present studies, specific siRNAs for COX-1 and -2 were used to evaluate the effects of COX-1 and -2 on myogenic differentiation. We employed our novel liquid chromatography-mass spectrometry/mass spectrometry (LC-MS/MS) method and a AA-targeted lipidomics method package, which is able to detect 87 compounds derived from AA, 18 eicosapentaenoic acid (EPA)-derived compounds, 16 docosahexaenoic acid (DHA)-derived compounds, and 11 ethanolamides for evaluating the changes in lipid profiling after knocking down COX-1 and -2 during myogenesis. In addition, based on the morphological changes induced by siRNA treatments, a customized skeletal muscle-targeted gene array [4] was used to identify genetic components regulated by COXs and LMs. We further linked these studies with functional measurements of intracellular calcium levels in myotubes, which is an essential surrogate for a host of skeletal muscle functions. Our results demonstrate that knocking down COXs has a significant effect on the synthesis of PGs in skeletal muscle cells. However, they function in a complex LM network not limited to PGs and have significant impacts on the levels of other LMs, such as oleoylethanolamide (OEA) and arachidonoylethanolamine (AEA), which are potentially new factors released from muscle for systemic metabolic regulation. Moreover, COXs play an important role in the regulation of gene expression of contractile apparatus and Ca^2+^ signaling, such as *Myh7*, *Cacna1s,* and *Itrp1*, which can be reflected in the changes observed in morphological and functional tests.

## 2. Results

### 2.1. Transfection with Specific siRNAs Targeting COX-1 or -2 Significantly Reduce the Expression Levels of COXs

**Figure 1:**
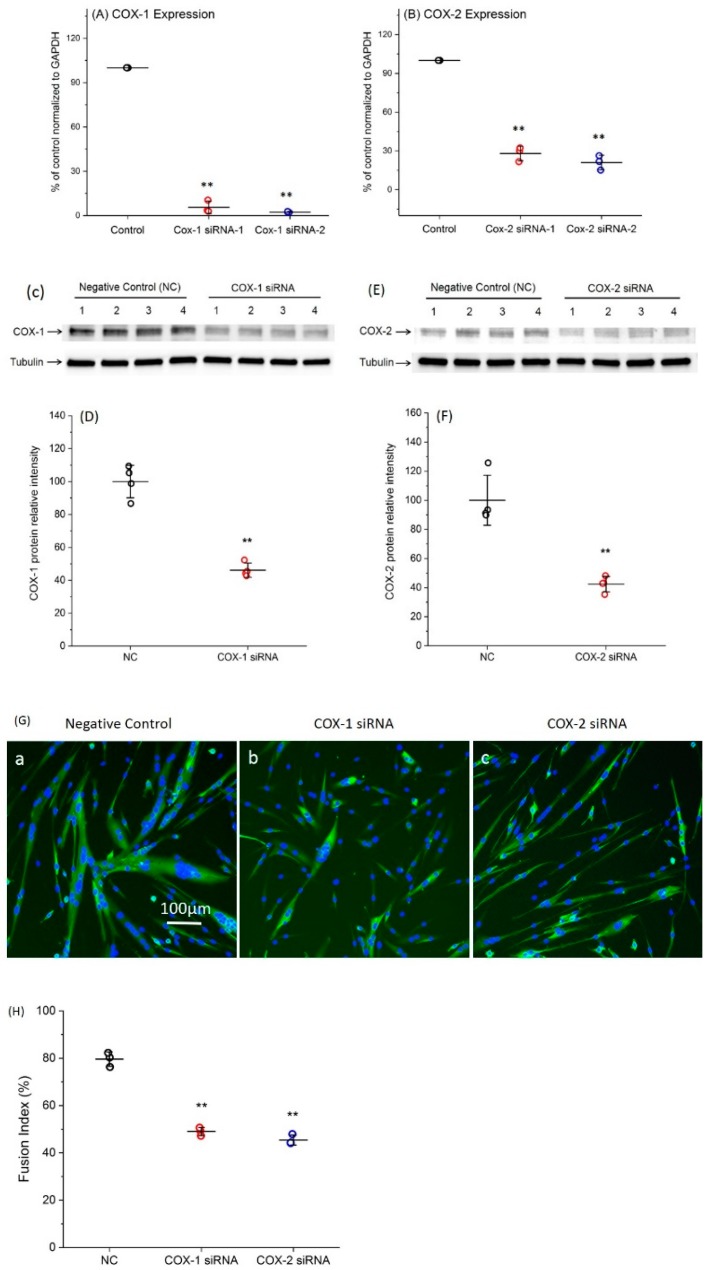
Verification of the high efficiency of COX-1 and COX-2 siRNA knockdown. (**A**) Knockdown efficiency of siRNAs targeting COX-1; (**B**) knockdown efficiency of siRNAs targeting COX-2; (**C**) COX-1 Western blot results after siRNA transfection for 48 h; (**D**) quantification of COX-1 Western blot results using ImageJ; (**E**) COX-2 Western blot results after siRNA transfection for 48 h; (**F**) quantification of COX-2 Western blot results using ImageJ; and (**G**) both COX-1 and COX-2 siRNA transfections inhibit primary myoblast myogenic differentiation. Morphological phenotypes observed after transfections with siRNAs. a: Negative control; b: COX-1 siRNA; and c: COX-2 siRNA. (**H**) Treatments with siRNAs significantly reduces fusion index. *n* = 3–4, ** *p* < 0.01 compared with NC.

Two siRNAs specific for each COX were transfected into primary myoblasts. Forty-eight hours after transfection, the total RNA was collected for quantitative RT-PCR to determine the changes in COX expression level. For each gene, both siRNAs efficiently decreased gene expression (Figure 1A,B). Since the siRNA-2 of COX-1 and -2 had higher levels of knockdown efficiencies, resulting in 97.2% and 79.3% downregulation of COX-1 and -2, respectively, compared with negative control (NC), they were used for all remaining experiments. In addition, the protein levels of COX-1 and -2 were shown around 55% reduction at 48 h post transfection with COX-1 or -2 siRNA (Figure 1C–F). Completed Western blot images are shown in supplementary Figure S1.

After 48 h transfection with COX-1 or -2 siRNA, significant morphological changes were observed in myotubes (Figure 1G). Quantified myogenic differentiation data showed that fusion index was reduced from 79.6% (NC) to 49% (COX-1 siRNA) and 45.4% (COX-2 siRNA), respectively (Figure 1H).

### 2.2. The Changes in Levels of Lipid Mediators after Knocking Down COX-1 or -2 Are not Limited to PGs and Thromboxane B_2_ (TXB_2_)

**Figure 2:**
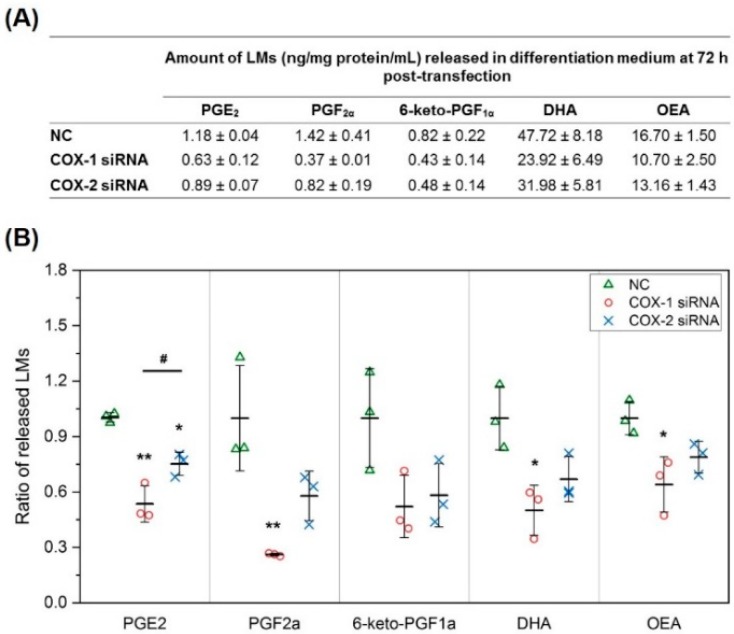
COX-1 and -2 knockdown reduces the levels of key lipid mediators released by primary muscle cells. (**A**) Absolute quantification of lipid mediators (LMs) released in differentiation medium (DM) from primary mouse myocytes/myotubes during differentiation; (**B**) ratio of LMs released in DM at 72 h post-transfection comparing COX-1 siRNA or COX-2 siRNA treatment with NC transfection. *n* = 3, * *p* < 0.05 and ** *p* < 0.01 compared with NC; ^#^ *p* < 0.05 compared with COX-1 siRNA.

To investigate the mechanisms responsible for the effect of COXs in skeletal muscle myogenesis, we first used our new lipidomics method to directly quantify 14 LMs selected from our preliminary studies, mostly AA metabolites through COX and other enzymes in cell differentiation medium (DM). Compared with blank medium, after differentiation for 72 h, the levels of PGE_2_, PGF_2α_, 6-keto-PGF_1α_ (stable metabolite of PGI_2_), DHA, and OEA in the medium increased significantly, suggesting that these LMs were released from myocytes/myotubes. We then further analyzed the effect of COXs on their production. Knocking down COX-1 using siRNA significantly reduced the levels of PGE_2_ and PGF_2α_ compared with NC, but had no significant effect on the levels of 6-keto-PGF_1α_. At the same time, knocking down COX-2 also showed a similar impact on PGE_2_ levels, but the effect was significantly less than knocking down COX-1. In addition to changes in LMs in AA pathway, COX-1 knockdown significantly reduced the concentration of DHA and OEA in the DM after 72 h (Figure 2). These data demonstrate that the functions of COXs are not limited to regulating the production of PGs from AA. The whole list of LMs identified in these experiments, including LMs with lower levels after 72 h differentiation compared with blank medium (LMs could be consumed by myocytes/myotubes during differentiation), is summarized in supplementary Figure S2.

### 2.3. COXs could Interact with LOXs to Regulate the Levels of Lipid Mediators

**Figure 3:**
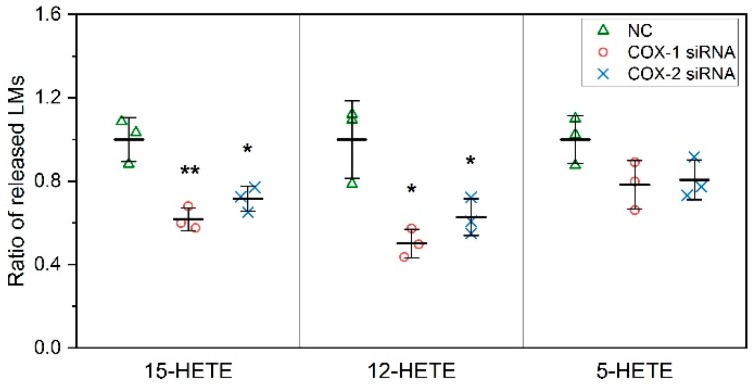
Knockdown of COXs reduces the levels of hydroxyeicosatetraenoic acids (HETEs) released by primary muscle cells. The levels of 12-HETE and 15-HETE, but not 5-HETE are significantly affected by the downregulation of gene expression of both COX-1 and COX-2. *n* = 3, * *p* < 0.05 and ** *p* < 0.01 compared with NC.

In addition to direct quantification for lipid mediators, lipidomic profiling of 158 lipid mediators in DM also was performed. Our results indicate that the levels of 12-Hydroxyeicosatetraenoic acid (12-HETE), a lipid mediator derived from the 12-LOX pathway, and 15-HETE, a lipid mediator derived from the 15-LOX pathway, significantly decreased after siRNA transfection targeting both COX-1 and -2. In contrast, the levels of 5-HETE, a lipid mediator derived from the 5-LOX pathway was not affected (Figure 3).

### 2.4. Supplement with LMs Improves Defective Myogenic Differentiation of Primary Myoblast Caused by Knocking Down COX-1 or -2

**Figure 4:**
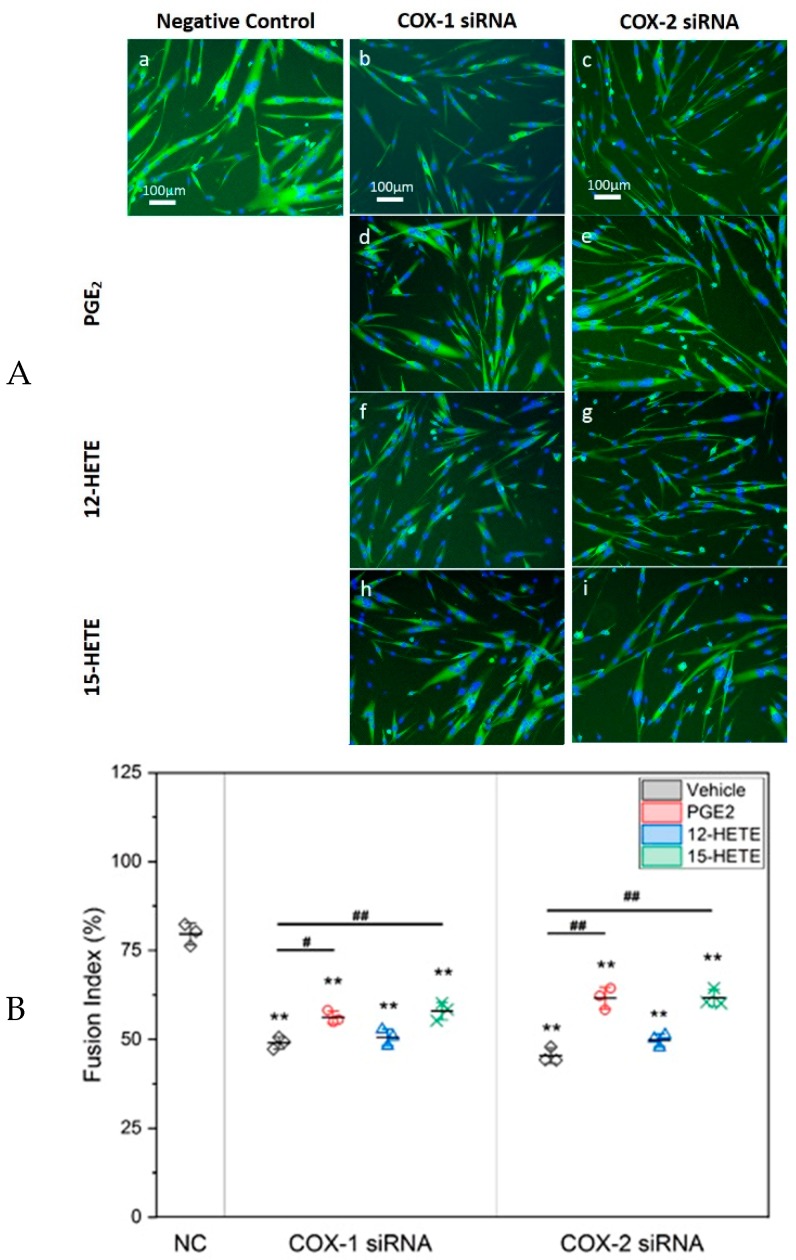
Treatment with PGE_2_ or 15-HETE partially recovers the impaired myogenesis induced by COX-1 or -2 knockdown. Panel (**A**): Representative fluorescence images of morphological changes of myotubes after siRNA transfection and supplement with LMs. Blue: DAPI (4′,6-diamidino-2-phenylindole) staining; green: MHC (myosin heavy chain) staining. Panel (**B**): Pretreatment with PGE_2_ and 15-HETE partially but significantly improved Fusion Index. *n* = 3, ** *p* < 0.01 compared with NC; ^#^ *p* < 0.05 and ^##^ *p* < 0.01 compared with COX-1 or -2 siRNA.

Based on the results of lipidomic analysis, to confirm that the effects on myogenic differentiation after knocking down COX-1 and -2 were through decreasing the production of LMs, three LMs, including PGE_2_, 12-HETE, and 15-HETE, were selected to determine whether replenishment with these LMs could improve defective myogenesis following transfection with siRNAs. Our results indicated that co-treatment with PGE_2_ or 15-HETE, but not 12-HETE, partially recovered the inhibition of both siRNAs used against COX-1 or -2 on myogenic differentiation. The fusion indexes increased significantly from 49% to 56.1% and 58.3% in culture treated with COX-1 siRNA, and from 45.4% to 59.8% and 62.3% in the COX-2 siRNA treated group, respectively. However, neither PGE_2_ nor 15-HETE brought the fusion index back to normal (negative control) level (Figure 4).

### 2.5. Results of Lipidomic Analysis of C2C12 Cells Show Similar Patterns as Primary Myoblasts

Following the studies of primary myoblasts, lipidomic analysis was performed in C2C12 cells. Since it is relatively easy to reach cell numbers high enough for reliable lipidomic analysis in C2C12 cell culture, we performed lipidomic studies in both cell culture media and cells.

**Figure 5:**
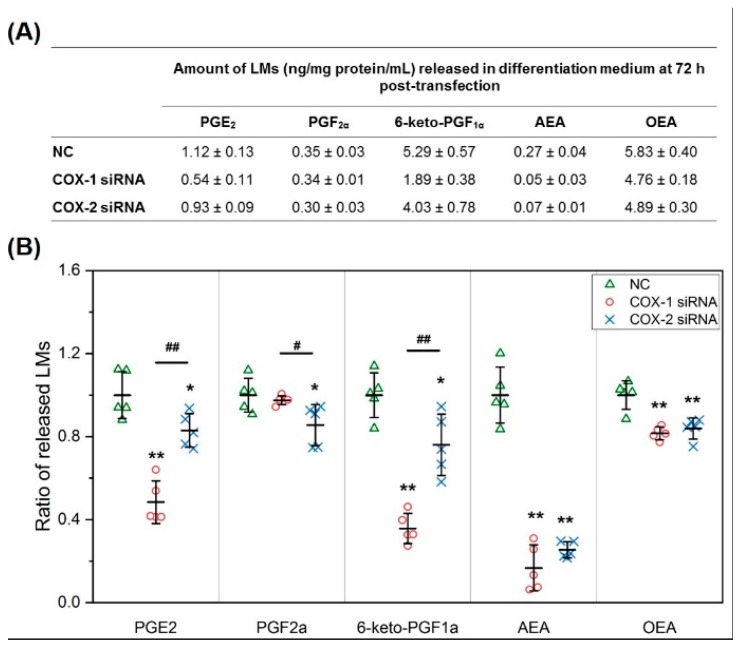
COX-1 or -2 knockdown reduces the levels of key lipid mediators released by C2C12 muscle cells. (**A**) Absolute quantification of LMs released in DM of C2C12; (**B**) ratio of LMs released in DM at 72 h post transfection comparing COX-1 siRNA or COX-2 siRNA treatment with NC transfection. *n* = 5, * *p* < 0.05 and ** *p* < 0.01 compared with NC; ^#^ *p* < 0.05 and ^##^ *p* < 0.01 compared with COX-1 siRNA.

In C2C12 cell culture media, similar to the results obtained in mouse primary myoblast cultures, PGs from the AA pathway, including PGE_2_, PGF_2α_, and 6-keto-PGF_1α_ (PGI_2_), were released from cells into media. In addition, AEA and OEA also were identified as LMs released by myocytes/myotubes during differentiation. Knocking down COXs significantly lowered the concentrations of PGE_2_, 6-keto-PGF_1α_, AEA, and OEA in media. COX-1 was more effective in modulating the concentrations of PGE_2_ and 6-keto-PGF_1α_, but COX-2 knockdown had more impact on the release of PGF_2α_. DHA was not a lipid mediator released by C2C12 cells during differentiation (Figure 5).

**Figure 6:**
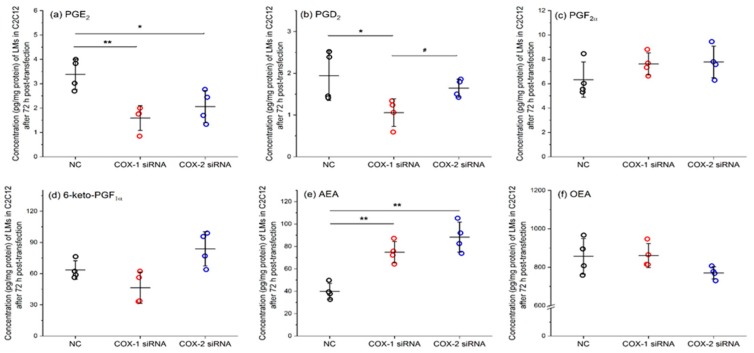
COX-1 or -2 knockdown alters the levels of key lipid mediators in C2C12 muscle cells. *n* = 4, * *p* < 0.05 and ** *p* < 0.01 compared with NC; ^#^ *p* < 0.05 compared with COX-1 siRNA.

In C2C12 cells, for LMs from AA pathway, downregulation of COXs significantly reduced the levels of PGE_2_, but had no effect on the levels of PGF_2α_ or 6-keto-PGF_1α_. Moreover, knocking down COX-1, but not COX-2, significantly lowered the concentration of PGD_2_. TXB_2_ was not detectable in C2C12 cells. Interestingly, knocking down COXs significantly increased the level of AEA in C2C12 cells, but had no effect on OEA levels (Figure 6). These results further confirm that the functional change in COXs affects a more complex network of LMs than just PGs and TXA_2_. The whole list of LMs identified in these studies using C2C12 cells is summarized in supplementary Figure S3 for cell culture medium and supplementary Figure S4 for C2C12 cells.

### 2.6. Changes in Gene Expression Profile after siRNA Transfection Targeting at COX-1 or -2

**Figure 7:**
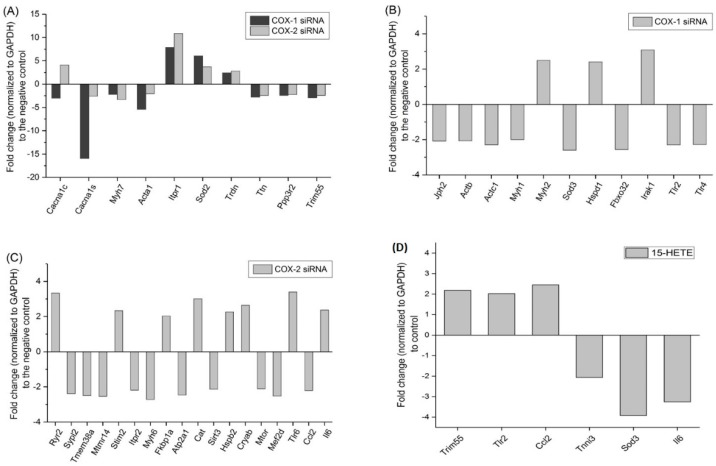
Knocking down COX-1 or -2 affects the expression of genes related with muscle structure and functions. (**A**) Genes affected by both COX-1 and COX-2 siRNA transfection; (**B**) genes affected by COX-1 siRNA transfection only; and (**C**) genes affected by COX-2 siRNA transfection only. (**D**) Changes in gene expression after treatment with 15-HETE for 48 h. Only genes with two-fold or greater changes, which are considered as significant changes, are listed.

Next, to study the genetic mechanism(s) related to the changes in lipid mediators after knocking down COX-1 or -2, a customized quantitative RT-PCR gene array, which includes 91 genes associated with cell myogenic differentiation, cell survival, Ca^2+^ signaling and homeostasis, cell metabolism, oxidative stress, and cell growth was performed [4]. After transfection with siRNAs, genes encoding components of contractile apparatus and Ca^2+^ signaling were significantly affected (Figure 7). *Myh7, Acta1*, *Ttn*, *Myh1,* and *Myh6* were downregulated by knocking down at least one of the COX isoforms. In contrast, the expression of *ITPR1* gene, which encodes the inositol 1,4,5-triphosphate (IP3) receptor 1, an important regulator of intracellular calcium signaling, was increased. However, knocking down COX-1 significantly reduced the expression levels of *Cacna1c* and *Cacna1s*, which are genes encoding subunits of voltage-sensitive, L-type calcium channel, and *Jph2*. The impact of COX-2 on calcium signaling is more complex, in addition to *Itpr1*, transfection with COX-2 siRNA also upregulated the expression of *Cacna1c*, *Ryr2*, and *Stim2*, but downregulated the expression of *Sypl2*, *Mtmr14*, *Tmem38a*, and *Itpr2*.

In addition, the changes in antioxidative genes (*Sod2*, *Sod3*, and *Cat*) and the upregulation of genes of heat shock protein family (*Hspd1*, *Hspb2,* and *Cryab*) suggest that the cells were under stress after transfection of the siRNAs.

The changes in gene expression after COXs downregulation could be directly related with the decreased levels of lipid mediators. We previously reported the effect of PGE_2_ on gene expression in muscle cells using our customized gene array [4]. In this study, using the same method, the changes in gene expression in primary mouse myoblast after 48 h of treatment with 15-HETE were determined. Genes encoding tripartite motif-containing protein 55 (Trim55), Toll-like receptor 2 (TLR2), and CC-chemokine ligand 2 (CCL2) were significantly upregulated after treatment with 15-HETE. *Trim55* is one of genes downregulated after transfection with either COX-1 or -2 siRNA, and the gene expression of *TLR2* and *CCL2* were significantly reduced by knocking down COX-1 and -2, respectively (Figure 7). These results support, at the genetic level, the partial recovery effect of myogenesis induced 15-HETE treatments shown in Figure 4.

### 2.7. Intracellular Calcium Homeostasis Measurement

Since there are significant changes in gene expression in the contractile apparatus and Ca^2+^ machinery, the measurement of intracellular calcium homeostasis was performed to identify functional changes in myotubes after siRNA transfection.

**Figure 8:**
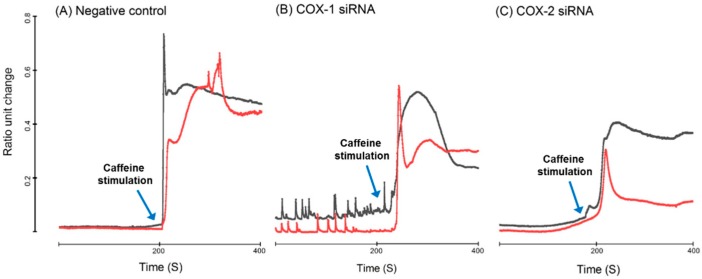
Representative Ca^2+^ transient of mouse primary myotubes loaded with Fura-2/AM in response to 20 mM caffeine (arrows). Treatment with COX-1 siRNA induced spontaneous Ca^2+^ oscillation with reduced response to caffeine stimulation. While Ca^2+^ oscillation was not observed in myotubes treated with COX-2 siRNA, their response to caffeine stimulation was further reduced. (**A**) Negative control; (**B**) COX-1 siRNA knockdown; and (**C**) COX-2 siRNA knockdown.

Both COX-1 and -2 siRNA transfection significantly altered the profile of intracellular calcium homeostasis in response to caffeine stimulation, but there was some difference between COX-1 and -2 knockdown. COX-1 siRNA treated myotubes demonstrated spontaneous cyclical transition in baseline fluorescence and a weaker response to caffeine stimulation compared to the negative control group. While COX-2 siRNA treated myotubes do not show cyclical oscillation in intracellular Ca^2+^ measurement, the amplitude of their responses to caffeine stimulation were further attenuated (Figure 8).

## 3. Discussion

COX-1 and -2 are the two most important enzymes in the synthesis of PGs and TXA_2_ from AA. Due to the comprehensive functions of PGs and TXA_2_ in physiological and pathological processes, COX-1 and -2 have been considered as important targets for the development of new therapeutics for disease [20,21]. In skeletal muscle, previous studies have shown that COXs, through the regulation of their AA metabolites, play important roles in muscle development, regeneration, and diseases [13,22].

To date, most studies concerned with the role of COXs in skeletal muscle have been focused on COX-2, the inducible form of COX. COX-2 increases during muscle regeneration after injury and during recovery from muscle atrophy [14]. Moreover, under normal conditions, the protein levels of both COX-1 and -2 are detectable in rat extensor digitorum longus (EDL) and soleus muscle [23]. Inhibition of COX-2 results in attenuated muscle growth during regeneration after injuries and reduced muscle hypertrophy in animal models [24]. At least part of the effects of COXs are thought to be mediated by the functions of their AA metabolites, which include PGE_2_, PGF_2α_, PGI_2_, PGD_2_, and TXA_2_. In skeletal muscle, due to their important functions in the regulation of myoblast proliferation and differentiation, and the function of inflammatory cells, PGE_2_ and PGF_2α_ have been considered major mediators of the effects of COXs [7,9]. PGI_2_ plays an important role in regulating the migration and fusion of muscle cells [25]. In contrast, treatment with PGD_2_ inhibited C2C12 myogenesis in vitro [26].

COX-2 induction during muscle regeneration occurs in the early acute inflammatory phase, which is involved in the recruitment of inflammatory cells, such as macrophages, activation of satellite cells, and myoblast proliferation [13]. However, administration of COX-2 inhibitor after acute inflammatory phase did not affect muscle regeneration and had no noticeable effect in undamaged muscles. These data suggest that COX-1, the constitutive isoform, may compensate for COX-2 and also plays a role in muscle regeneration and in the maintenance of normal muscle functions. By downregulating COX-1 and -2 expression in mouse primary myoblast using siRNAs, we compared the functionalities of these enzymes in myogenic differentiation. Our results demonstrated that knocking down both COX-1 and -2 significantly inhibited myogenesis. However, these two enzymes may have different functions in myogenic differentiation, based on the morphological phenotypes after the transfection of siRNAs.

To our knowledge, there is currently no systematic study comparing the functionalities of COX-1 and -2 during myogenesis in terms of the production of AA metabolites and other aspects important for myoblast migration, proliferation, differentiation, and fusion, such as lipid profiling and intracellular calcium homeostasis.

Our data provide evidence supporting previous findings that PGE_2_ and PGF_2α_ could be two major mediators from the AA/COX pathway in skeletal muscle. Moreover, the changes in PGs and TXB_2_ clearly indicate that COX-1 plays a critical role in the stages from myoblast to fusion in myogenesis. In addition to affecting the production of PGs and TXs, reduced COXs functionalities also affected the levels of AA metabolites through LOX pathways. 5- and 12/15 LOXs are the LOX isoforms utilizing AA as substrate to generate 5-, 12-, and 15-HETE. Our results demonstrated that reduced COX-1 or -2 expression significantly decreased the levels of 12- and 15-HETE, but had no effect on 5-HETE. These results suggest that COXs could interact with LOX to regulate the production of lipid mediators from AA. 12/15 LOX shares some function with COXs, such as the regulation of inflammatory cytokines. In animal studies, deletion of 12/15 LOX prevents the early onset of inflammation caused by a high-fat diet [27] and denervation-induced muscle atrophy [28]. On the other hand, the same genetic manipulation resulted in exaggerated inflammation and tissue damage in arthritis, and disruption of the translocation of glucose transporter type 4 in cardiac and skeletal muscle. Our results suggest that COX-1 and -2 could function indirectly on LMs by altering the metabolism of AA by LOXs. This could be the first evidence of the interaction between COXs and LOXs in skeletal muscle.

Recently, skeletal muscle has been recognized as an endocrine tissue. Factors released from muscles, such as β-aminoisobutyric acid (BAIBA), a muscle metabolite, can act as endocrine factors to crosstalk with bone, adipose tissue, and other tissues or organs [29,30]. In our study, besides PGs, OEA was also identified as a factor released by skeletal muscle, a metabolite derived from omega-9 fatty acid, oleic acid. BAIBA, via activation of peroxisome proliferator-activated receptor α (PPARα), transient receptor potential vanilloid type-1 (TRPV1), and G protein coupled receptor GPR119 regulates fat catabolism, food intake, and glucose homeostasis [31,32,33]. In soleus muscle, OEA enhanced the oxidation of fatty acid, but had no significant effect on glucose metabolism [34]. Currently, feeding status and enzymes directly responsible for OEA synthesis or degradation, such as N-acyl transferase and fatty acid amide hydrolase [35], are major factors affecting the OEA level. Our results demonstrated that COXs in skeletal muscle could be an important factor regulating the OEA level. AEA is another candidate lipid mediator acting as a myokine, because it has important functions in metabolic regulation and anti-inflammatory effects through activating TRPV1 and cannabinoid receptors, respectively [36], and in our studies is regulated by the activities of COXs in skeletal muscle. These data could help to expand the pool of myokines and provide new insight for explaining the beneficial effect of exercise.

The regulatory function of skeletal muscle on metabolism is closely related with its status, especially functionality status. After transfection with siRNAs targeting COXs, the development of myotubes is inhibited. Corresponding with this phenotype, genes encoding components of contractile apparatus and cytoskeleton, including *Myh2*, *Myh7*, *Acta1*, *Actb*, and *Actc1*, were also significantly affected. Appropriate cytoskeletal remodeling, which also is related to the assembly of the contractile apparatus, is critical for migration, cell-to-cell recognition, and fusion of myoblasts/myocytes [37]. The changes in gene expression of the contractile apparatus and cellular structural components suggest that COXs are important for assembly of contractile apparatus and cytoskeleton. Moreover, after knocking down COX-1 or -2, functional tests using the measurement of intracellular Ca^2+^ homeostasis in myotubes was performed. Our results indicate that intracellular Ca^2+^ signaling was defective after downregulation of COXs. COX-1 siRNA treated myotubes demonstrated spontaneous cyclical transition in baseline fluorescence and a weaker response to caffeine stimulation. These phenomena could have been resulted from the changes in gene expression of Ca^2+^ machinery. *Cacna1c* and *Cacna1s* are genes encoding subunits of the voltage-sensitive, L-type Ca^2+^ channel, which plays a critical role in gating intracellular Ca^2+^ movement [38]. Significant downregulation of these two genes after knocking down COX-1 could lead to the dysfunction of voltage-sensitive, L-type Ca^2+^ channels, which could be the major reason for the detectable spontaneous Ca^2+^ transients in myotubes. While COX-2 siRNA treated myotubes did not show similar changes in intracellular Ca^2+^ measurement, the amplitude of their responses to caffeine stimulation were further attenuated. Gene expression of *Sypl2* (*Mg29*) and *Mtmr14* significantly decreased after COX-2 knockdown. Previous findings from our group have confirmed that knocking down these genes causes defective Ca^2+^ signaling in skeletal muscle [39,40]. These changes, along with downregulation of *Tmem38a*, a gene encoding trimeric intracellular cation channel type A, which is important for maintenance of rapid intracellular calcium release [41], could contribute to the attenuated response upon caffeine stimulation.

The changes in gene expression after COX-1 and -2 siRNA transfection could be modulated through decreasing levels of 15-HETE. Treatment with 15-HETE significantly increased the expression of *Trim55, TLR2,* and *CCL2*. *Trim55*, also called muscle-specific RING finger protein 2 (*MuRF2*), was downregulated after knocking down COX-1 or -2. This gene has been shown to be important for the organization of cytoskeleton and contractile machinery in muscle. A reduced *Trim55* expression level led to delayed myoblast fusion, defective contractile function, and deformation of Z- and M-bands, suggesting that Trim55 is an adaptor for tubulin, titin, and myosin, which has an important impact on structural and functional aspects in muscle [42,43]. *TLR2* and *CCL2* were genes downregulated by knocking down COX-1 and -2, respectively. They are important components in inflammatory responses, which play essential roles in immune responses, muscle regeneration after injuries and muscle atrophy [44,45]. During endurance training, TLR2 signaling mediates the activation of mitogen-activated protein kinase (MAPK) and nuclear factor κB (NF-κB) induced by extracellular nonesterified fatty acids [46]. One the other hand, muscle atrophy after immobilization is closely related with oxidative stress and inflammation through the activation of TLR2 [47]. *CCL2* might be one of the targets of TLR2 signaling in skeletal muscles. Peptidoglycan, an agonist of TLR1 and TLR2, significantly induced *CCL2* expression in C2C12 myotubes [48]. Polymorphisms of *CCL2* are associated with muscle adaption and muscle damage response caused by exercise [49,50]. Research concerned with TLR2 and CCL2 in muscle has been focused on their functions in recruiting immune cells, such as monocytes, during muscle recovery from injury, which involves cell migration and cell adhesion [51,52]. Myoblast migration and adhesion are important steps for differentiation and fusion. Our results imply that COXs-15-HETE signaling could be important for pre-fusion events in myogenesis. Another interesting finding is that transfection with COX-2 siRNA significantly increased the expression of *interleukin-6 (IL-6)*, which was reversed when myoblasts were treated with 15-HETE. IL-6 is a multi-functional factor in skeletal muscle. It can stimulate satellite cell proliferation [53], but chronic exposure to IL-6 led to muscle atrophy [54], which is supported by the previous report that inhibition of IL-6 signaling attenuated muscle atrophy in tail suspension model through the downregulation of atrophy-related genes, such as atrogin-1 [55].

Collectively, these studies provide new insights into the regulation of LMs in skeletal muscle and their crucial function for muscle cell homeostasis.

## 4. Materials and Methods

### 4.1. Cell Culture

#### 4.1.1. Myoblast Isolation and Culture

Isolation of primary myoblasts was performed as previously described [7]. Primary myoblasts were isolated from hind limb muscles of 5 months old C57BL/6 mice. Collected muscles were minced and digested using 0.1% pronase (EMD Millipore, Temecula, CA, USA). Isolated cells (fibroblasts and myoblasts) were maintained and expanded in collagen-I (Corning, Corning, NY, USA) coated T-75 flask in growth medium (GM) consisted of Ham’s F-10 (Corning), 20% fetal bovine serum (FBS, Atlanta Biologicals, Flowery Branch, GA, USA), 5 ng/mL basic recombinant human fibroblast growth factor (Promega, Fitchburg, WI, USA), 100 µg/mL streptomycin (Thermo Scientific, Rockford, IL, USA), and 100 U/mL penicillin G (Thermo Fisher Scientific, Waltham, MA, USA) for 3 to 4 weeks for purification. For differentiation, purified myoblasts were plated on E-C-L (Millipore)-coated 6-well plates at ~200,000 cells/well and differentiated in DM for 48 or 72 h.

#### 4.1.2. C2C12 Cells

C2C12 cells were cultured as previously described [56]. Briefly, cells [American Type Culture Collection (ATCC), Manassas, VA, USA] were cultured in complete growth medium [CGM, high-glucose Dulbecco’s Modified Eagle Medium (DMEM, Corning) with 10% fetal bovine serum, plus 100 U/mL penicillin and 100 μg/mL streptomycin (Thermo Fisher Scientific)], at 37 °C and 5% CO_2_. C2C12 myoblasts were maintained at 70–80% confluence and passaged one or two times before being used in experiments.

To initiate differentiation, CGM was replaced by differentiation medium (DM) containing high-glucose DMEM, 2.5% horse serum (Hyclone Laboratories Inc, Logan, UT, USA), 100 U/mL penicillin, and 100 μg/mL streptomycin.

### 4.2. siRNA Transfection

For primary mouse myoblasts, cells were seeded at ~200,000 cells/well in 6-well plates in primary GM, then differentiated overnight before being transfected with 10nM siRNAs, including negative control siRNA and siRNAs targeting COX-1 or -2 [Integrated DNA Technologies (IDT), Coralville, IA, USA]. Lipofectamine RNAiMAX (Thermo Fisher Scientific) was used as a transfectant following the instructions from the manufacturer.

For recovery experiments with LM supplements, including PGE_2_, 12-HETE, and 15-HETE, primary myoblasts were treated with 50 nM of each LM for 2 h in fresh DM before being transfected with siRNAs.

For C2C12 cells, cells grew in CGM until 80–90% confluence in 6-well plates, then differentiated overnight before being treated with siRNAs, as described in primary myoblast experiments.

### 4.3. Quantitative Real-Time PCR (qRT-PCR)

Total RNA was extracted from primary myoblasts using Direct-zol RNA MiniPrep (Zymo Research, Irvine, CA, USA) according to the manufacturer’s instruction, and was quantified in a Nanodrop 1000 spectrophotometer (Thermo Fisher Scientific). An aliquot of RNA sample (0.5–1 µg) with the A260/280 nm absorbance ratio of 1.8 or above was reverse transcribed in a 20 µL reaction volume using a protoscript II first strand cDNA synthesis kit (New England Biolabs, Ipswich, MA, USA).

The RT-PCR reaction mixture contained 2 µL cDNA, 12.5 µL of the RT^2^ SYBR Green/Rox PCR master mix (Qiagen, Germantown, MD, USA), 0.4 µL of primer pairs (10 µM) and 10.1 µL of RNase free water to a complete reaction volume of 25 µL. qRT-PCR was performed using Step-One Plus TM RT-PCR System (Thermo Fisher Scientific), and results were normalized to the reference gene GAPDH. Primers used in the experiments include: 1) COX-1: Forward: 5′-TGCCCATGGAGACCAGAAGAAGTT-3′; Reverse: 5′-ATGGGTGTGGAGAAATGGCTCAGT-3′; 2) COX-2: Forward: 5′-ATGACTGGCTGGTGCATCTCATCT-3′; Reverse: 5′-ACTTGCCCTCACGGACAATGTAGT-3′; 3) GAPDH: Forward: 5′-TGCGATGGGTGTGAACCACGAGAA-3′; Reverse: 5′- GAGCCCTTCCACAATGCCAAAGTT-3′.

The customized gene array was previously developed by our laboratory in collaboration with Qiagen and is now commercially available from Qiagen (Item No.: CAPM09345C, Germantown, MD, USA) [4]. Experiments were performed according to the instructions from the manufacturer. Data were uploaded and analyzed by specific software from Qiagen. Changes in gene expression were considered significant when change was two-fold or greater.

### 4.4. Protein Sample Preparation and Western Blotting

Muscle cells cultured in 6-well plates were washed 3 times with ice-cold Dulbecco’s phosphate buffered saline (PBS) before being lysed by RIPA buffer [1× Tris-buffered saline (TBS), 1% Nonidet P-40, 0.5% sodium deoxycholate, 0.1% SDS, 0.004% sodium azide] (Sigma-Aldrich, St. Louis, MO, USA) with 1% cocktail of proteinase and phosphatase inhibitors (Sigma-Aldrich). Lysates were then collected and incubated in ice for 30 min, followed by centrifugation at 16,000× *g* for 20 min at 4 °C. Supernatants were collected for protein assay.

Protein assay was performed using Micro BCA Protein Assay Kit (Thermo Fisher Scientific) according to the manufacturer’s instructions. Protein samples then were mixed with 4× Western blot loading buffer (Bio-Rad, Plano, TX, USA) and denatured at 100 °C for 5 min.

For Western blots, ~30 µg of total proteins were fractionated by 4–15% Mini Protean TGX gels (Bio-Rad) and transferred to polyvinylidene difluoride (PVDF) membranes (Bio-Rad). Membranes were blocked in 5% non-fat dry milk in 1× TBS with 0.1% Tween 20 (TBST) for 1 h at room temperature (RT), followed by incubation with antibodies COX-1 (1:1000, Cell Signaling Technology, Inc, Danvers, MA, USA) and β-tubulin (1:1000, Cell Signaling Technology, Inc, Danvers, MA, USA) in 5% bovine serum in TBST or COX-2 antibody (1 μg/mL, R&D systems, Minneapolis, MN, USA) in 5% non-fat dry milk at 4 °C overnight. HRP-conjugated goat anti-rabbit (For COX-1 and β-tubulin, 1:10,000, Jackson ImmunoResearch, West Grove, PA, USA) or HRP-conjugated rabbit anti-goat (For COX-2, 1:5000, Thermo Fisher Scientific) secondary antibodies were then applied to membranes for 1 h at RT. After five 5-min washes in TBST, Clarity Max ECL Western blotting substrates (Bio-Rad) or Super Signal West Femto substrate (Thermo Fisher Scientific) were used to detect the signal by ChemiDoc MP imaging system (Bio-Rad).

### 4.5. Immunohistochemistry

After differentiation, cells in 6-well plates were fixed in 10% neutral buffered formalin solution (NBF, Sigma-Aldrich) for 15 min. After removal of NBF, cells were washed 4 times with PBS, followed by permeabilization with 0.1% Triton X-100 in PBS for 15 min. Cells were then incubated with myosin heavy chain (MHC) fluorescein-conjugated antibody (1:100, R&D Systems) overnight at 4 °C. After 3 washes with PBS, DAPI (1:1000, Sigma-Aldrich) was added for 10 min incubation at room temperature. Images were taken with Olympus IX50 system using software cellSens Dimension 1.15 (Olympus Corp., New Orleans, LA, USA).

### 4.6. LC-MS/MS

#### 4.6.1. Sample Preparation for Lipidomics Analysis

Briefly, cells from four wells of 6-well plates were harvested after experiments and transferred into 1.0 mL of ice-cold 80% methanol in water (*v*/*v*) to perform homogenization using the TissueLyser II homogenizer (Qiagen) at the frequency of 30/sec, in 6 × 30 s bursts, and 20 s in between to avoid high temperature. An aliquot (20 µL) of cell homogenates was saved separately for future protein content measurement by BCA (bicinchoninic acid) assay (Thermo Scientific, Rockford, IL, USA). The remaining homogenate from each cell sample was added to 10 µL of IS mixture stock solution (5 µg/mL for AA-d_8_, 2 µg/mL for DHA-d_5_ and EPA-d_5_, and 0.5 µg/mL), then agitated on ice in the dark for 1–2 h. For cell culture media, 1 mL of culture media sample was mixed with 1.5 mL of ice-cold methanol and 10 µL of IS mixture stock solution, then agitated on ice in dark for 15 min. After incubating the homogenate or culture media sample on ice, samples were centrifuged at 6000× *g* at 4 °C for 10 min to remove any precipitated proteins. All LM standards and isotope-labelled LM internal standards were purchased from Cayman Chemical Co (Ann Arbor, MI, USA). Formic acid (reagent grade, ≥95%) was obtained from Sigma-Aldrich. HPLC-MS grade acetonitrile, water, methanol, and ethanol were purchased from J.T. Baker (Phillipsburg, NY, USA).

Both cell and culture media samples were cleaned and concentrated by Solid Phase Extraction (SPE) before being injected into the LCMS. Before loading samples to the preconditioned SPE cartridges (Strata-X 33 µm polymeric reversed phase, Phenomenex, Torrance, CA, USA), 4 or 6 mL of ice-cold 0.1% formic acid was added in the supernatant from cell or medium sample, respectively, to fully protonate the LM species. Once the sample had been totally loaded, cartridges were washed with 1 mL of 0.1% formic acid followed by 1 mL of 15% (*v*/*v*) ethanol in water to remove excess salts. Then the LMs from the SPE sorbent bed were eluted by methanol. Solvents were removed using an Eppendorf^®^ 5301 concentrator centrifugal evaporator (Eppendorf, Hauppauge, NY, USA), and the dried extracts stored at −80 °C for future LC-MS/MS analysis.

#### 4.6.2. LC-MS/MS Conditions

All components of LC-MS/MS system are from Shimadzu Scientific Instruments, Inc. (Columbia, MD, USA). LC system was equipped with four pumps (Pump A/B: LC-30AD, Pump C/D: LC-20AD XR), a SIL-30AC autosampler (AS), and a CTO-30A column oven containing a 2-channel six-port switching valve. The LC separation was conducted on a C8 column (Ultra C8, 150 × 2.1 mm, 3 µm, RESTEK, Manchaca, TX, USA) along with a Halo guard column (Optimize Technologies, Oregon City, OR, USA). The MS/MS analysis was performed on Shimadzu LCMS-8050 triple quadrupole mass spectrometer. The instrument was operated and optimized under both positive and negative electrospray and multiple reaction monitoring modes (+/− ESI MRM). The settings of flow rate and gradient program for the LC system as well as MS/MS conditions are recommended by a software method package for 158 lipid mediators (Shimadzu Scientific Instruments, Inc., Columbia, MD, USA) and further optimized following our previously published quantification method [57]. Briefly, the optimized conditions are as follows: Interface voltage, 4.0 kV; interface temperature, 275 °C; DL temperature, 275 °C; heating block temperature, 400 °C; drying gas (N_2_), 10 L/min; nebulizing gas (N_2_), 3 L/min; heating gas (Air), and 10 L/min; CID gas (Ar), 230 kPa. The acquisition was divided into multiple segments. The *m*/*z* transitions and their tuning voltages were selected based on the best MRM responses from the instrumental method optimization software. All analyses and data processing were completed on Shimadzu LabSolutions V5.91 software (Shimadzu Scientific Instruments, Inc., Columbia, MD, USA).

### 4.7. Intracellular Ca^2+^ Measurements

Intracellular Ca^2+^ measurements were performed as previously described [58]. A Photon Technology International (PTI) imaging system was used to measure intracellular Ca^2+^ homeostasis. Myotubes imaged were loaded with 2 μM Fura-2 AM (Thermo Fisher Scientific) for 30 min at 37 °C, followed by RT de-esterification for 15 min. Only cells that had an initial ratio below 1.0, indicating healthy and not Ca^2+^-overloaded myotubes, were selected for application of 20 mM caffeine (Thermo Fisher Scientific) with a perfusion system (Bioscience Tools, San Diego, CA, USA). Ratiometric analysis (350/375 nm excitation ratio; 510 nm emission) was performed using software PTI EasyRatioPro 2 (HORIBA, Edison, NJ, USA). These experiments were repeated 5 times and at least 6 myotubes were tested on each experiment.

### 4.8. Statistical Analysis

Three to five independent replicates were performed for each experiment, except for the customized gene array study. One-way ANOVA with post hoc Tukey’s test was performed for data analysis. Results were expressed as mean ± SD. Differences were considered significant at *p* < 0.05.

## 5. Conclusions

In conclusion, by using lipidomic analysis, our data have provided new insights regarding the functions of COXs in skeletal muscle. COX-1 may play a major role in the step from myoblast to fusion in myogenic differentiation. Its effect could also be related with the alteration in AA/LOX pathway. Further studies on the lipid mediators from AA/COX pathway, and the interactions between COXs and LOXs will advance the knowledge of COX and related lipid signaling in skeletal muscle and other tissues, which could benefit the development of new treatments for inflammation related diseases in skeletal muscle and other tissues.

## Supplementary Materials

Supplementary materials can be found at https://www.mdpi.com/1422-0067/20/18/4326/s1.
