# Supplementary material for: Multi-Staged Regulation of Lipid Signaling Mediators during Myogenesis by COX-1/2 Pathways
Source: Int J Mol Sci. 2019 Sep 4;20(18):4326. doi: 10.3390/ijms20184326 (PMC6769623; doi:10.3390/ijms20184326)
Supplement: Supplementary file 1 [file ijms-20-04326-s001.pdf]

Supplementary Figure Legends:

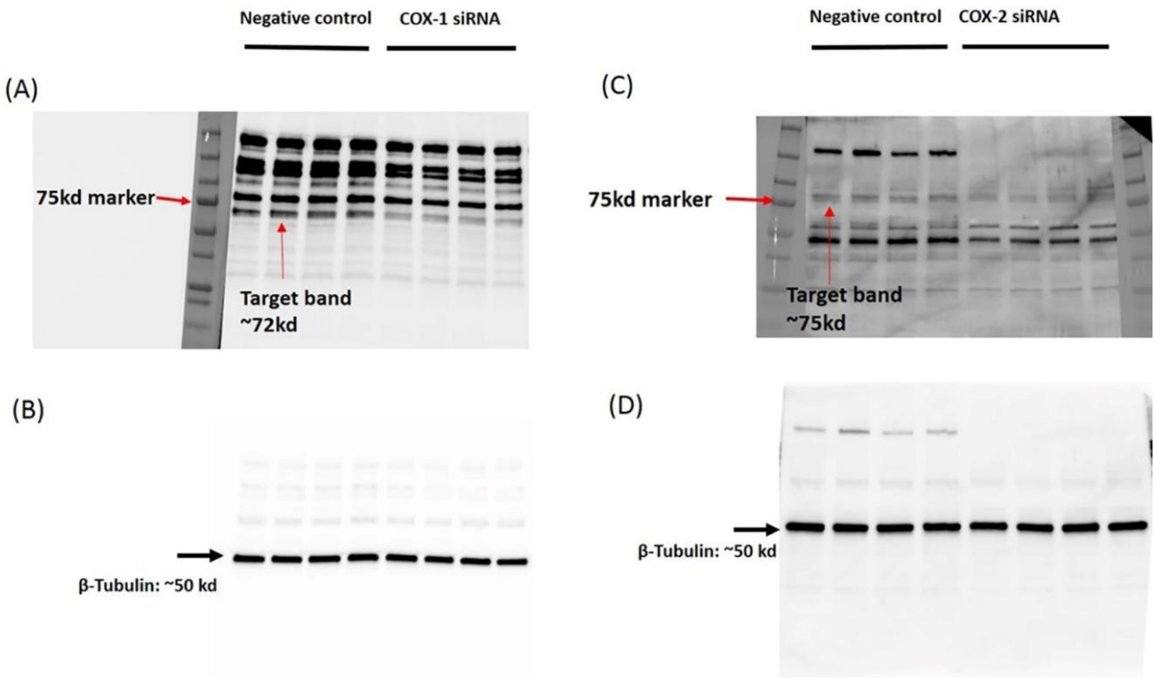

Figure S1: The completed images of Western blot results. (A) COX-1 Western blot results and corresponding loading control β-tubulin (B); (C) COX-2 Western blot results and corresponding loading control β-tubulin (D).

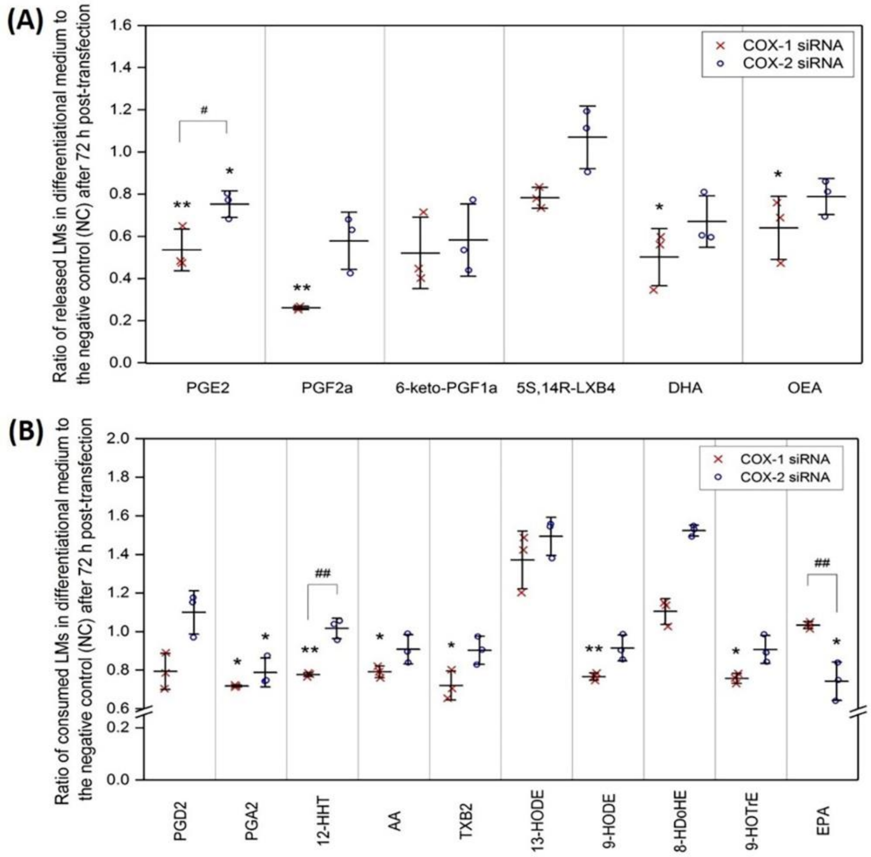

Figure S2: **Results of lipodomic quantification in DM for primary myoblast after COX-1 and -2 knockdown.** (A) LMs released by myocytes/myotubes into DM after 72 h

differentiation; (B) LMs consumed by myocytes/myotubes into DM after 72 h differentiation (concentrations were lower than blank medium after differentiation). n=3, *p<0.05 and **p< 0.01 compared with negative control; # p<0.05 and ## p<0.01 compared with COX-1 siRNA.

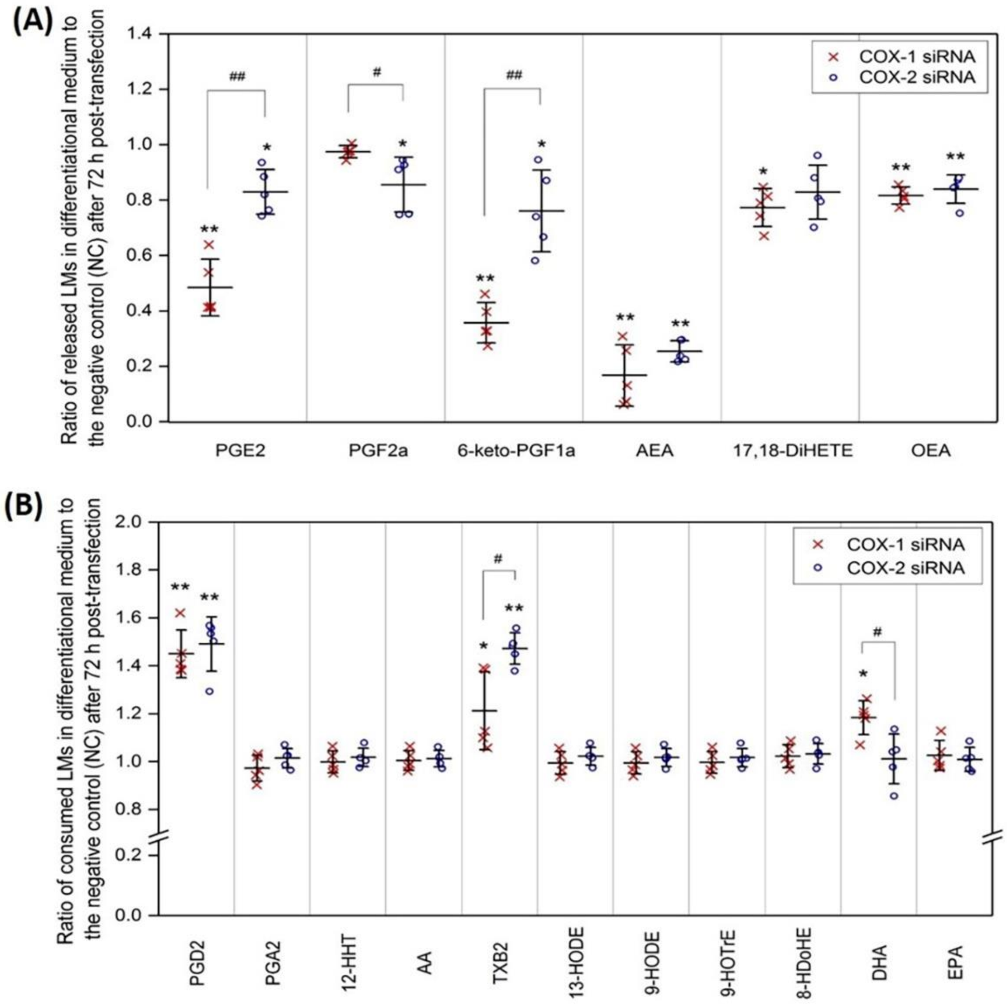

Figure S3: **COX-1 and -2 knockdown leads to specific changes in LMs in DM of C2C12 culture.** (A) LMs released by C2C12 cells/myotubes into DM after 72 h differentiation; (B) LMs consumed by C2C12 cells/myotubes into DM after 72 h differentiation (concentrations were lower than blank medium after differentiation). n=5, *p<0.05 and **p< 0.01 compared with negative control; # p<0.05 and ## p<0.01 compared with COX-1 siRNA.

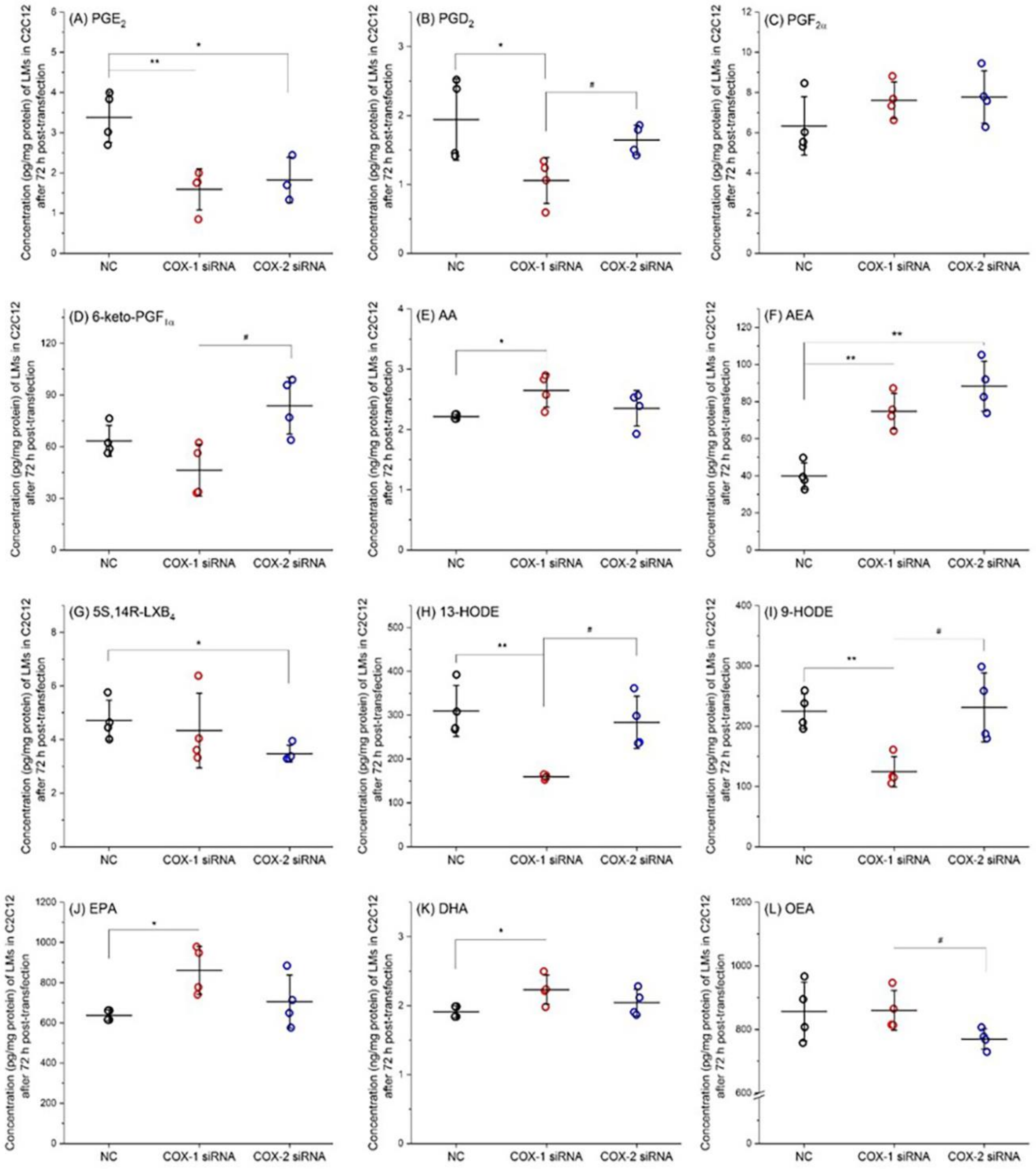

Figure S4: **COX-1 and -2 knockdown leads to specific changes in LMs in C2C12 muscle cells.** n=4, **p*<0.05 and ***p*< 0.01 compared with NC; # *p*<0.05 compared with COX-1 siRNA.
